# Large nonlinear dielectric behavior in BaTi_1−x_SnxO_3_

**DOI:** 10.1038/s41598-017-07192-x

**Published:** 2017-07-27

**Authors:** Pengrong Ren, Zicheng Liu, Qian Wang, Biaolin Peng, Shanming Ke, Huiqing Fan, Gaoyang Zhao

**Affiliations:** 1 Shaanxi Province Key Laboratory for Electrical Materials and Infiltration Technology, School of Materials Science and Engineering, Xi’an University of Technology, Xi’an, 710048 P.R. China; 2 School of Physical Science & Technology, Guangxi Key Laboratory for Relativistic Astrophysics, Guangxi University, Nanning, 530004 P.R. China; 3 College of Materials Science and Engineering, Shenzhen University, Shenzhen, 518060 P.R. China; 4 State Key Laboratory of Solidification Processing, School of Materials Science and Engineering, Northwestern Polytechnical University, Xi’an, 710072 P.R. China

## Abstract

BaTi_1−x_Sn_x_O_3_ (BTSn, 0 ≤ x ≤ 0.30) ceramics were prepared by both the conventional sintering (CS) and sparking plasma sintering (SPS). Composition, temperature and grain size dependences of the nonlinear dielectric behaviors were systematically studied. BTSn was found to have especially large tunability (≥90%), which is larger than most other Pb-free systems, and is comparable to Pb-based relaxors. The high dielectric tunability in BTSn is attributed to its specific domain structures. Besides, temperature dependent tunability of BTSn presents a dispersed behavior and the dispersion is enhanced with the increase of Sn^4+^ concentrations, which is explained by the compositional fluctuation model.

## Introduction

With the rapid development of tunable devices, nonlinear dielectric materials that have high tunability and low dielectric loss have been investigated extensively. Of the numerous perovskite-structured materials that exhibit variable permittivity under an external *dc* bias field, solid solutions, such as Ba_1−x_Sr_x_TiO_3_ (BST)^1^ and BaTi_1-y_Zr_y_O_3_ (BZT)^2^, have been widely studied. By adjusting the Sr and Zr ions concentrations in BST and BZT, respectively, for example, x = 0.6 or y = 0.35^3, 4^, the dielectric permittivity of the materials have been found to be highly tunable at the ambient temperature. In addition to normal ferroelectrics, some relaxor ferroelectric materials have also been reported to have abnormally large dielectric tunability, such as Pb(Mg_1/3_Nb_2/3_)_0.88_Ti_0.12_O_3_^5^, Pb(Ni_1/3_Nb_2/3_)_0.5_Zr_0.15_Ti_0.35_O_3_^6^ and Pb_0.8_Ba_0.2_ZrO_3_^7^. The tunable dielectric properties of these relaxor ferroelectric ceramics are attributed to the presence of the “extrinsic” polar clusters as well as “intrinsic” lattice phonon polarization. The high dielectric tunability and low dielectric loss under *dc* electric field in relaxor ferroelectric ceramics is achieved by controlling the size of the polar clusters, which merged into polar nanoregions (PNRs)^8^.

Recently, researchers have shown great interest in developing environmentally-friendly lead-free relaxors, in particular those based on BaTiO_3_. The relaxor behavior in these kinds of materials has been in materials with a heterovalent cation substitution as well as in isovalently substituted-solid solutions such as BaTi_1−x_Sn_x_O_3_ (BTSn). A systematic investigation of the Sn^4+^-substitution on the phase transition behavior has been reported^9–13^. And tunabale dielectric properties of BTSn were reported by Chen^14^ and Lu^15^. However, the majority of researches have focused on identifying the roles of dynamic/static PNRs on phase transition behavior^16, 17^. By contrast, research efforts towards the effects of domain structures (the morphology of domain, domain size, PNRs) on electric field-dependent nonlinear dielectric behaviors remain limited. Meanwhile, in addition to the contribution of PNRs, some researchers have argued that grain size has effects on the dielectric behavior of ferroelectric materials^18, 19^. However, the effect of grain size on the nonlinear dielectric behaviors remains controversial.

In the present work, BaTi_1−x_Sn_x_O_3_ (BTSn, 0 ≤ x ≤ 0.30) solid solutions with a wide compositional range, from the normal ferroelectricity to the diffused phase transition (DPT) state, have been studied. The BTSn ceramics have grain size of 10~20 μm and 1 μm when prepared by the conventional sintering (CS) or sparking plasma sintering (SPS), respectively. The effects of domain structure and grain size on the nonlinear dielectric behavior in BTSn are investigated.

## Experimental procedure

Samples of BaTi_1−x_Sn_x_O_3_ with x = 0, 0.02, 0.04, 0.11, 0.15, 0.20 and 0.30 (labelled as BT, BTSn02, BTSn04, BTSn11, BTSn15, BTSn20 and BTSn30, respectively) were prepared by a solid state reaction using BaCO_3_ (99.9%), TiO_2_ (99.9%) and SnO_2_ (99.9%) as starting reagents. The raw powders were dried at 180 °C, weighed according to the nominal stoichiometric ratio and then ball milled in ethyl alcohol using zirconia balls for 12 h. The resulting calcined powder was cold-isostatic-pressed under a pressure of 200 MPa to give a disk with a diameter of 10 mm and a thickness of 1 mm. The BTSn ceramics were then sintered at 1450 °C for 2 h under air. In order to study the effects of grain size on nonlinear dielectric behavior, BTSn15 ceramics were sintered by an SPS method at 1175 °C for 10 min. The phase structures of the BTSn ceramic powders were determined by X-ray powder diffraction (XRD; D/Max2550VB+/PC, Rigaku, Tokyo, Japan) using Cu-Kα1 radiation and linear position-sensitive detector. Raman scattering experiments of the ceramics were performed using an instrument (LabRAM HR800, Horiba JobinYvon, Lyon, France) in a backward scattering geometry where the exciting source was a 514.5 nm line from an argon ion laser. The microstructure of the samples were observed using a scanning electron microscope (SEM, DSM 950, Zeiss, Oberkochen,Germany) operated at 15 kV. Transmission electron microscopy (TEM) samples were prepared by including grinding, cutting, dimpling, and ion milling. The dimpled disks were annealed at 200 °C for 2 h to minimize any artifacts that may have been introduced during mechanical thinning. A transmission electron microscope (Tecnai F30, FEI, Hillsboro, OR) at 200 kV accelerating voltage was used to analyze the disks.

A metal-insulator-metal (MIM) capacitor was formed in order to characterize the electric properties. To form this capacitor, the sintered pellets were polished, coated with silver electrodes and fired at 600 °C for 30 min. The dielectric properties of BTSn ceramics were investigated over the range of 200 °C to −100 °C (cooling rate 3 °C/min) using a precision LCR meter (E4294A, Agilent, Santa Clara, CA, USA) connected with a temperature controller (TP94, Linkam, Surrey, UK). Frequencies from 100 Hz to 1 MHz at a signal level of 0.5 V/mm were used for the measurement. The dielectric tunability properties were investigated at 25 °C by using an automatic component analyzer (TH2818, Tonghui, Changzhou, China) at 10 kHz. A blocking circuit was adopted to protect the analyzer from applied bias voltages. The external bias field was applied in steps of 1 kV/cm. And the temperature dependent tunability was measured by put a sample on a heater and the temperature is controlled from room temperature to 150 °C. Ferroelectric hysteresis loops were measured at room temperature using a ferroelectric tester (TF2000, aixACCT, Aachen, Germany).

## Results and Discussions

**Figure 1:**
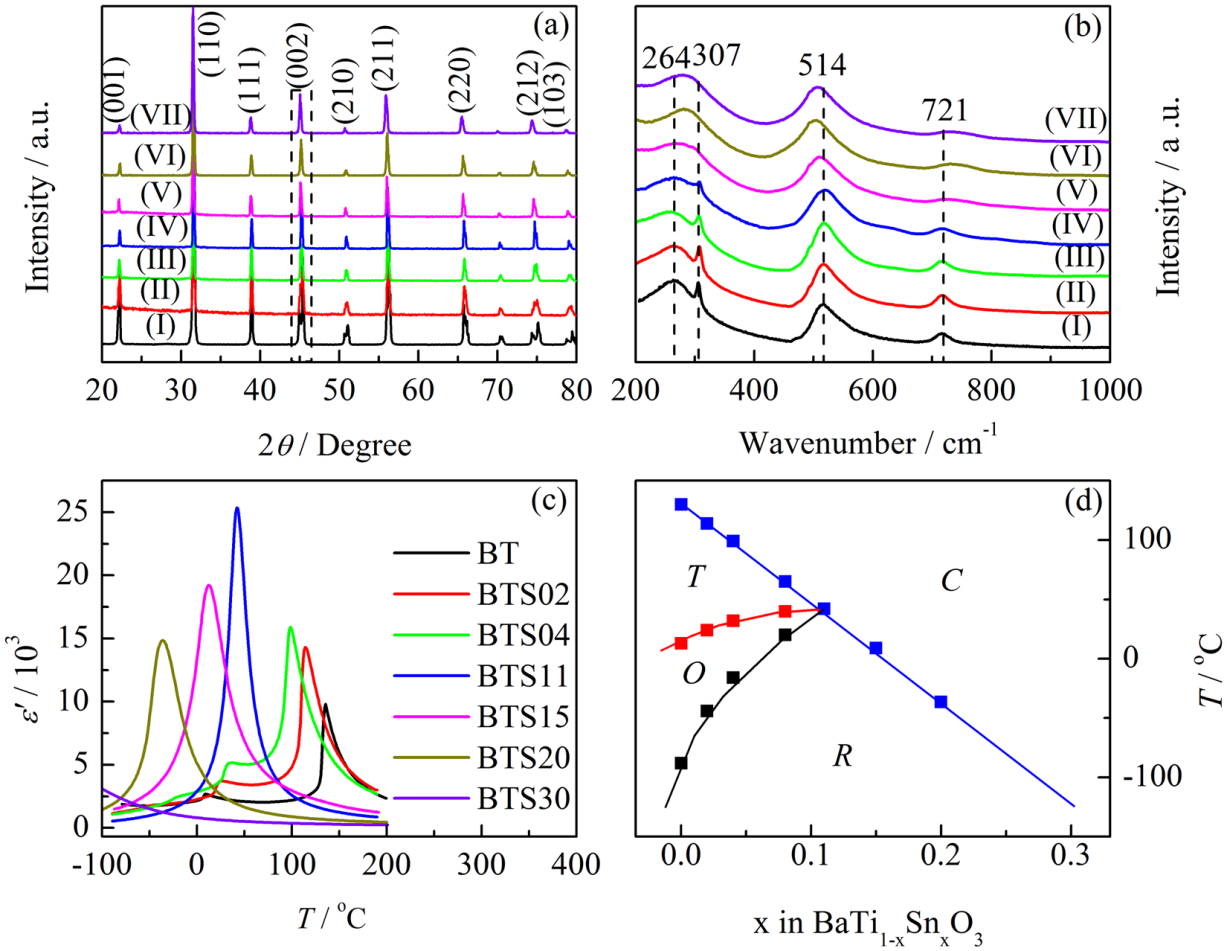
(**a**) XRD patterns and (**b**) Raman spectra of BTSn ceramics; (**c**) the temperature dependence of dielectric permittivities of BTSn ceramics; (**d**) the phase diagram of BTSn ceramics with the Sn doping level ranging from 0% to 30%.

Figure 1(a) shows XRD patterns of BTSn ceramics recorded at room temperature. The XRD patterns clearly show the formation of pure perovskite structure without any secondary phases. The split characteristic and the relative intensity of (002)/(200) peaks at about 45° indicates that at room temperature BTSn has a tetragonal phase with x ≤ 0.04, while it turns into a unique peak with x ≥ 0.11, suggesting a rhombohedral or cubic phase is obtained with x ≥ 0.11 at room temperature. The Raman spectra in Fig. 1(b) show similar results. The peak at 307 cm^−1^, which is a characteristic peak of tetragonal BaTiO_3_^20^, diminishes with increasing Sn content, and disappears when x ≥ 0.11. The temperature dependence of the dielectric permittivity recorded at 10 kHz and 0.5 V during the cooling process from 200 to −100 °C for the seven BTSn compositions is shown in Fig. 1(c). Three permittivity anomalies show that the samples undergo three phase transitions with decreasing temperature, i.e., cubic-tetragonal (*C-T*), tetragonal-orthorhombic (*T-O*) and orthorhombic-rhombohedral (*O-R*) for BT, BTSn02 and BTSn04. In contrast, BTSn11, BTSn15 and BTSn20 display only one phase transition from the cubic phase to rhombohedra phase at 42, 13 and −36 °C, respectively^12^. The phase transition of BTSn30 occurs beyond the measured temperature range. For better illustration, a rough phase diagram of BTSn is shown in Fig. 1(d). The *C-T*, *T-O* and *O-R* phase transition lines gradually merge and the four phases eventually meet together, at a quasi-quadruple point around 42 °C with a composition of BTSn11. This is in agreement with the reported literature values^12^. At room temperature (25 °C), BTSn11 is in a ferroelectric state, while BTSn15 and BTSn20 are in a paraelectric state. As a result, each of them shows different dielectric behaviors, and each of these behaviors is studied in the following.

**Figure 2:**
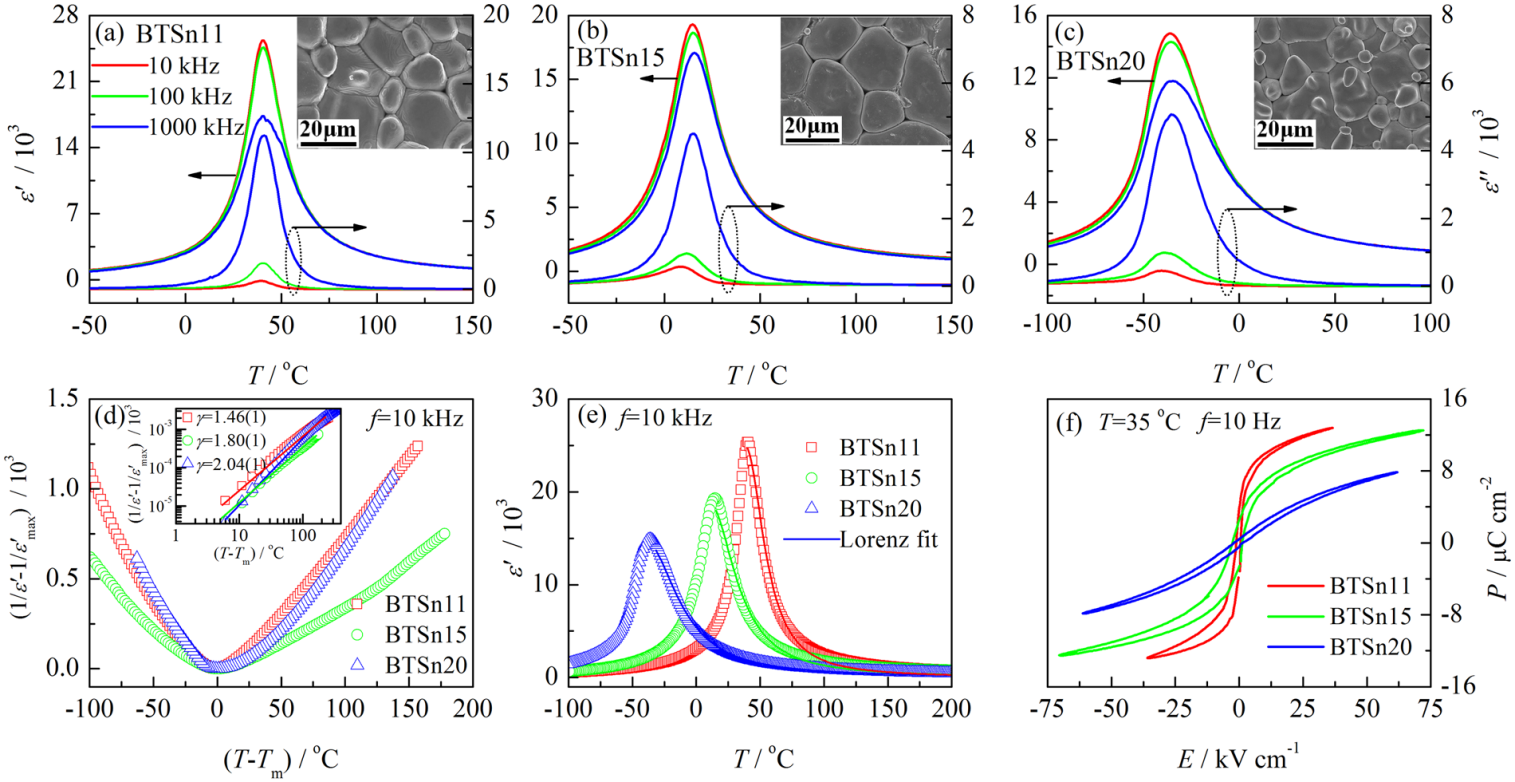
(**a–c**) The temperature dependences of the real (*ε*′) and imaginary parts (*ε*″) of the complex dielectric permittivities of the BTSn11, BTSn15 and BTSn20 ceramics. The insets in (**a**–**c**) are SEM images of the surface of the ceramics; (**d**) the temperature dependences of the inverse dielectric permittivity. In the inset of (**d**), the same dependences are plotted on a log-log scale, where the values of the parameter were estimated in accordance with Eq. (1) for *T* − *T*m > 100 °C; (**e**) the temperature dependences of the dielectric permittivity of the BTSn ceramics. The solid lines are fitting curves derived by using Eq. (2); (**f**) *P*-*E* loops of the BTSn11, BTSn15 and BTSn20 ceramics measured at 32 °C and 10 Hz.

Figure 2(a–c) show the temperature dependences of the real (*ε′*) and imaginary parts (*ε*″) of the complex dielectric permittivities of BTSn11, BTSn15 and BTSn20 measured at different frequencies. With increasing Sn content, the peak of the dielectric permittivitity becomes progressively more diffuse, but the peak position does not change. This behavior is denoted as DPT in order to differentiate it from relaxor behavior. Although DPT behavior has been observed in a number of systems, the interpretations of the nature of DPT behavior are complicated and controversial^21, 22^. In BTSn ceramics, substitution of Ti^4+^ by Sn^4+^ results in the random breaking of Ti–O bonds because Sn^4+^ does not go off-center. Since the ionic radius of Sn^4+^ is larger than Ti^4+^, Sn^4+^ has less free space to shift within the oxygen octahedron. Spatial fluctuations of defective bonds lead to fluctuations of polar correlations, therefore precursor clusters are created and the Curie peak is broadened^10^. The insets in Fig. 2(a–c) show the microstructure of BTSn11, BTSn15 and BTSn20, respectively. Each sample has a dense microstructure with grain sizes 10~20 μm.

In order to quantitatively describe the broad Curie peak, the temperature dependences of the dielectric permittivity were fit by using the Curie-Weiss and Lorenz law. Figure 2(d) shows the temperature dependence of the inverse dielectric permittivity as measured at *f* = 10 kHz plotted against a reduced temperature scale. Evidently, within a certain temperature range above *T*_m_, 1/*ε*′ is not linear as is predicted by the Curie-Weiss law. Therefore, we adopted an empirical relationship to describe the temperature dependence of the dielectric permittivity^23, 24^

1 $$1/{\varepsilon }^{^{\prime} }=1/{\varepsilon }_{max}^{^{\prime} }+{[(T-{T}_{m})/{C}^{^{\prime} }]}^{\gamma }$$

where *C*′ and *γ* are constants. The value of *γ* is regarded as an indicator of the diffuseness of the phase transition. When *γ* = 1, Eq. (1) indicates a normal ferroelectric behavior and when *γ* = 2 it indicates a so-called DPT behavior. It is apparent in Fig. 2(d) that with an increase in Sn content, there is a systematic increase in *γ* in a fixed temperature range (as shown in the inset of Fig. 2(d)).

Bokov *et al*.^25^ recently proposed a Lorenz formula to describe the dependence of the dielectric permittivity on temperature at T > T_m_ in relaxors,

2 $${\varepsilon }_{A}^{^{\prime} }/{\varepsilon }^{^{\prime} }=1+{(T-{T}_{A})}^{2}/2{\delta }^{2}$$

where the temperature (*T*_A_) and the magnitude (*ε′*_A_) of the Lorenz height generally differ from the *T*_m_ and *ε′* of the experiments. The parameter *δ* is frequency independent at high enough frequencies and characterizes the diffuseness of the peak. The behavior of the dielectric permittivity peak on the high temperature side predicted by Eq. (2) has been found in a number of relaxor ferroelectrics, as well as in the prepared BTSn11, BTSn15 and BTSn20. Excellent fits are achieved above *T*_m_. The result of the fitting is shown in Fig. 2(e) by a solid line and the best-fit parameters are listed in Table 1. It should be noted that the increase of *δ* with increasing Sn concentration in BTSn, from 12.1(2) to 21.5(2) indicates that there is an increase in degree of diffuseness of the dielectric peak. As a result, the dielectric permittivity peak is more diffuse at larger x. However, no relaxor behavior was observed for any of the prepared ceramics, including BTSn20. The observed peculiarities of the temperature-dependent are in good agreement with the literature^8^.

**Table 1:** The parameters obtained from fitting by Eqs (1) and (2).

Samples	*f*/kHz	*T*_*m*_/°C	${\varepsilon }_{{\rm{\max }}}^{{}^{{\boldsymbol{^{\prime} }}}}$	*γ*	*T*_A_/°C	${\varepsilon }_{{\rm{A}}}^{{}^{{\boldsymbol{^{\prime} }}}}$	*δ*
BTSn11	10	42	25355	1.46(1)	38.8(2)	24882(9)	12.1(2)
BTSn15	10	13	19302	1.80(1)	10.0(4)	19000(8)	17.3(3)
BTSn20	10	−36	14844	2.04(1)	−40.1(3)	14778(8)	21.5(2)

*P*-*E* loops of BTSn11, BTSn15 and BTSn20 were measured at 32 °C and 10 Hz and are shown in Fig. 2(f). At 32 °C, BTSn11 is in a ferroelectric state, while BTSn15 and BTSn20 are in a paraelectric state. However, all the samples show *P*-*E* loops. As Sn concentration is increased in BTSn, the saturated polarization and remnant polarization decrease. A very slim *P*-*E* loop was obtained in BTSn20. There is also no obvious hysteresis phenomena but the nonlinearity (with *P*_r_ = 0.41 μC/cm^2^ and *E*_c_ = 1.78 kV/cm), suggesting the presence of PNRs, due to the fact that macroscopic domain switching gives rise to hysteresis, while microscopic domain switching leads to a nonlinear response to the external electric field.

**Figure 3:**
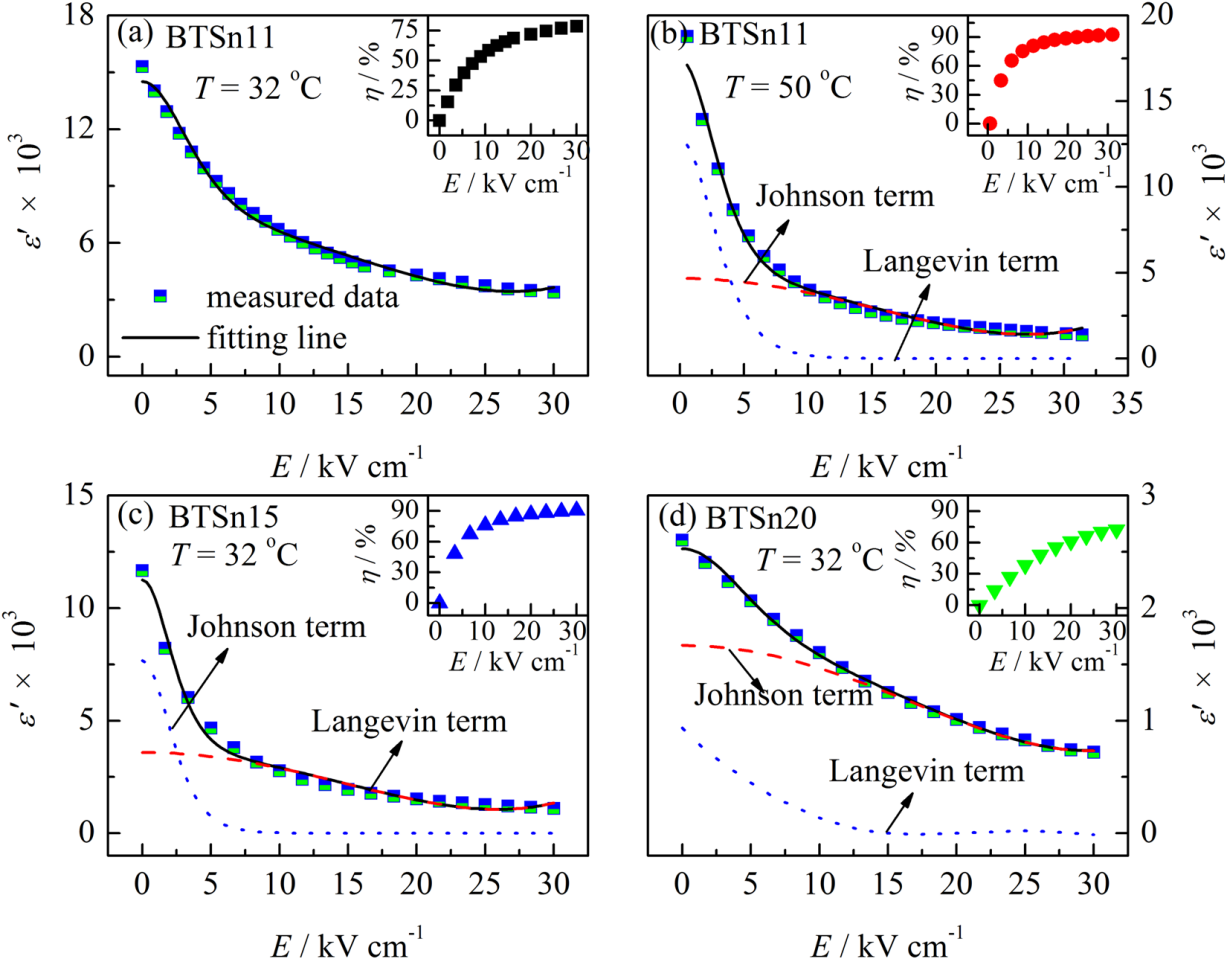
(**a–d**) Dielectric permittivity as a function of *dc* electric field of BTSn11, BTSn15 and BTSn20 from bias electric field measurement taken at 10 kHz and at the temperature of 32 °C and 50 °C. The data of BTSn11 measured at 32 °C were fitted by Eq. (3); the data of BTSn11 measured at 50 °C, BTSn15 and BTSn20 were fitted by Eq. (4); and Johnson term and Langevin term were simulated by the fitting parameters. The insets in (**a**–**d**) are dielectric tunability of BTSn11, BTSn15 and BTSn 20 as a function of the electric field.

Figure 3 illustrates permittivity as a function of *dc* electric field from bias electric field measurement taken at 10 kHz and room temperature. The measurements were made by stepwise increasing bias electric field from 0 to 30 kV/cm. With the electric field increasing, the permittivitties of BTSn11, BTSn15 and BTSn20 monotonously but nonlinearly decrease. Traditionally, in the ferroelectric state, the permittivity as a function of electric field can be described by using a Johonson relation:

3 $${\varepsilon }^{{\rm{^{\prime} }}}(E)={\varepsilon }_{r}(0)/{\{1+\lambda [{\varepsilon }_{0}{\varepsilon }_{r}{(0)}^{3}{E}^{2}]\}}^{1/3}$$

and in the paraelectric state, the permittivity as a function of electric field can be described by using a multipolarization-mechanism model^26^:

4 $${\varepsilon }^{{\rm{^{\prime} }}}(E)={\varepsilon }_{r}(0)/{\{1+\lambda [{\varepsilon }_{0}{\varepsilon }_{r}{(0)}^{3}{E}^{2}]\}}^{1/3}+({P}_{0}x/{\varepsilon }_{0}){[\cos {\rm{h}}(Ex)]}^{-2}$$

In Eq. (4) the first term represents the “intrinsic” Johnson contribution (the lattice polarization induced from the LGD theory) and the second term represents the “extrinsic” Langevin contribution (the reorientational polarization). *λ* is the anharmonic coefficient, which is a constant. *P*_0_ is the effective polarization of one cluster, *L* is the cluster size, *k*_B_ is the Boltzmann constant, *T* is the temperature and *x* = *P*_0_ *L* ^3^/*k*_B_ *T*.

At 32 °C BTSn11 is in a ferroelectric state, therefore, its permittivity as a function of the electric field was fitted by using Eq. (3), as shown in Fig. 3(a). The measured data have a good agreement with the fitting line. BTSn11 at 50 °C, BTSn15 and BTSn20 at 32 °C are all in a paraelectric state. Therefore, their permittivities as a function of the electric field were described by using Eq. (4), and the corresponding Johnson term and Langevin term were simulated by using the fitting parameters, as shown in Fig. 3(b–d). The fitting plots are in good agreement with *dc* bias dependent permittivity, with *R* ^2^ being 0.993, 0.990 and 0.999 for BTSn11, BTSn15 and BTSn20, respectively. The fitting parameters λ, *L* and *P*_0_ are listed in Table 2. The values of *λ* are in an order of 10^10^ magnitude for BTSn11, BTSn15 and BTSn20, respectively, which are in agreement with the results of Bi-doped SrTiO_3_ (6.0 × 10^10^ V m^5^ C^−3^)^26^ and Ba_0.6_Sr_0.4_TiO_3_ (2.9 × 10^10^ V m^5^ C^−3^)^27^. Polar clusters of BTSn11, BTSn15 and BTSn20 are 6.7(1), 9.3(1) and 4.1(1) nm in size, respectively. Besides, *P*_0_ are 4.1(1), 2.8(1) and 0.50(1) μC cm^−2^ for BTSn11, BTSn15 and BTSn20, respectively. These polarizations are close to the remnant polarizations obtained from the aforementioned *P*-*E* loops, as shown in Fig. 2(f).

**Table 2:** The fitting parameters by using Eq. (4): anharmonic coefficient (λ), cluster size (*L*) and effective polarization (*P*_0_), the contribution of the Johnson term (*t*_J_) and Langevin term (*t*_L_), the overall tunability (*η*) of BTSn ceramics.

Samples	*λ/*V m^5^ C^−3^	*L*/nm	*P*_0_/μC cm^−2^	*t*_J_/%	*t*_L_/%	*η*/%
BTSn11*	9.2(1) × 10^10^	6.7(1)	4.1 (1)	28.42	71.58	92.6
BTSn15	7.5(1) × 10^10^	9.3(1)	2.8 (1)	21.27	78.73	90.5
BTSn20	2.1(1) × 10^10^	4.1(1)	0.50 (1)	49.72	50.28	72.3
BTSn15-SPS	9.7(1) × 10^10^	5.5(1)	4.1 (1)	35.74	64.26	78.1

^*^Parameters of BTSn11 are obtained based on the dielectric tunability data measured at 50 °C. Parameters of other samples are obtained based on the dielectric tunability data measured at 32 °C.

Tunability (*η*) is usually calculated by using the following expression:

5 $$\eta =\frac{{\varepsilon }^{^{\prime} }(0)-{\varepsilon }^{^{\prime} }(E)}{{\varepsilon }^{^{\prime} }(0)}\times 100{\rm{ \% }}$$

where *ε*′(0) and *ε*′(*E*) represent the dielectric permittivity at zero and a certain electric field, respectively. The insets in Fig. 3 show the tunability of BTSn11, BTSn15 and BTSn20 as a function of electric field measured at 32 °C and 50 °C, with *f* = 10 kHz and with the maximum electric field of 30 kV/cm. At 32 °C, BTSn15 exhibits the maximum tunability (90.5%), which is larger than most Pb-free systems, for example, BST^3^ and BZT^4^, and even comparable with the Pb-based relaxors, such as Pb(Mg_1/3_Nb_2/3_)_0.88_Ti_0.12_O_3_ and Pb_0.8_Ba_0.2_ZrO_3_^5–7^. BTSn11 has larger tunability at 50 °C, which is up to 92.6%.

The overall tunability can be ascribed to the contribution of the Johnson term (*t*_J_) and the Langevin term (*t*_L_), which are quantified by Eq. (6) and (7), respectively:

6 $${t}_{J}=\frac{{\varepsilon }_{J}^{{}^{{\rm{^{\prime} }}}}(0)-{\varepsilon }_{J}^{{}^{{\rm{^{\prime} }}}}(E)}{{\varepsilon }^{{\rm{^{\prime} }}}(0)-{\varepsilon }^{{\rm{^{\prime} }}}(E)}\times 100{\rm{ \% }}$$

7 $${t}_{L}=\frac{{\varepsilon }_{L}^{{}^{{\rm{^{\prime} }}}}(0)-{\varepsilon }_{L}^{{}^{{\rm{^{\prime} }}}}(E)}{{\varepsilon }^{{\rm{^{\prime} }}}(0)-{\varepsilon }^{{\rm{^{\prime} }}}(E)}\times 100{\rm{ \% }}$$

*ε*′_J_(0), *ε*′_L_(0), *ε*′_J_(*E*) and *ε*′_L_(*E*) are dielectric permittivity of the simulated Johnson term and Langevin term at zero and a certain electric field, respectively. The calculated *t*_J_ and *t*_L_ are listed in Table 2. As shown in Table 2, BTSn15 has the maximum Langevin contribution (78.73%), which suggests that the “extrinsic” polarization plays a major role on its nonlinear dielectric behavior. For normal ferroelectrics such as PbTi_1−x_Zr_x_O_3_ (PZT) and BaTiO_3_ (BT), the “extrinsic” polarization is usually derived from the rotation of domain and domain-wall motion. However, For relaxors such as Pb(Mg_1/3_Nb_2/3_)O_3_ (PMN)^28^ and BaTi_1−x_Zr_x_O_3_ (BZT)^8^ the “extrinsic” polarization is mainly from the contribution of nanometer polar clusters. BTSn15 is located at an intermediate state between “normal” ferroelectric and relaxor. Therefore, it is probably that the rotation of domain, domain-wall motion as well as polar clusters co-contribute on its nonlinear dielectric behavior.

**Figure 4:**
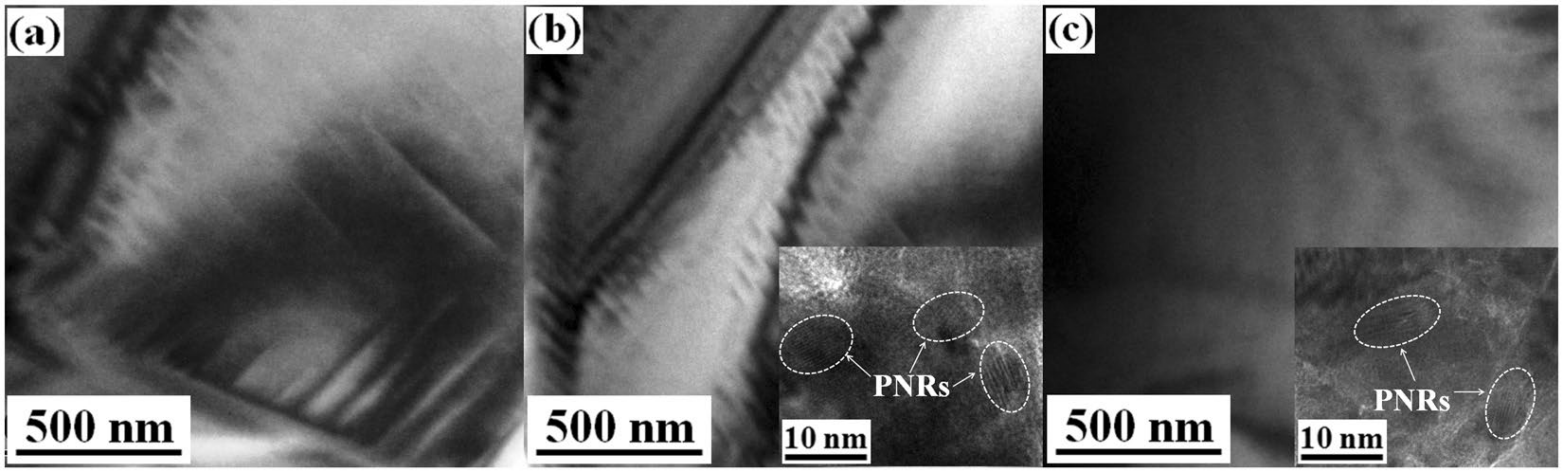
TEM images of (**a**) BTSn11, (**b**) BTSn15 and (**c**) BTSn20 at room temperature.

Figure 4 is typical TEM image of BTSn11, BTSn15 and BTSn20 at room temperature. In BTSn11, lamellar-like domains are observed. The width between two domain boundaries is about 150 nm. However, in BTSn15, lamellar-like but slim domains that are ~50 nm in size appear, meanwhile PNRs with a size of 10 nm are present and coexist with the lamellar-like domains, as shown in Fig. 4(b). BTSn20 does not exhibit lamellar-like domains, while PNRs with a size of 10 nm are observed. As the domain size is proportional to the square root of domain wall energy^29^, slim domains with reduced domain wall energy can easily respond to external excitations (e.g., mechanical force or electric field). For this reason, BTSn15 can be easily excited by electric field and its permittivity suddenly decreases when applied a small electric field, as shown in Fig. 3(c). Therefore BTSn15 has the maximum Langevin contribution and has a large tunability at room temperature. Such a phenomenon was also validated in both lead-based and lead-free piezoceramics. Zhang^30^ reported that nanodomains (50 ± 2 nm) could be assembled into domain stripes after poling, thus benefit the high piezoelectric properties.

**Figure 5:**
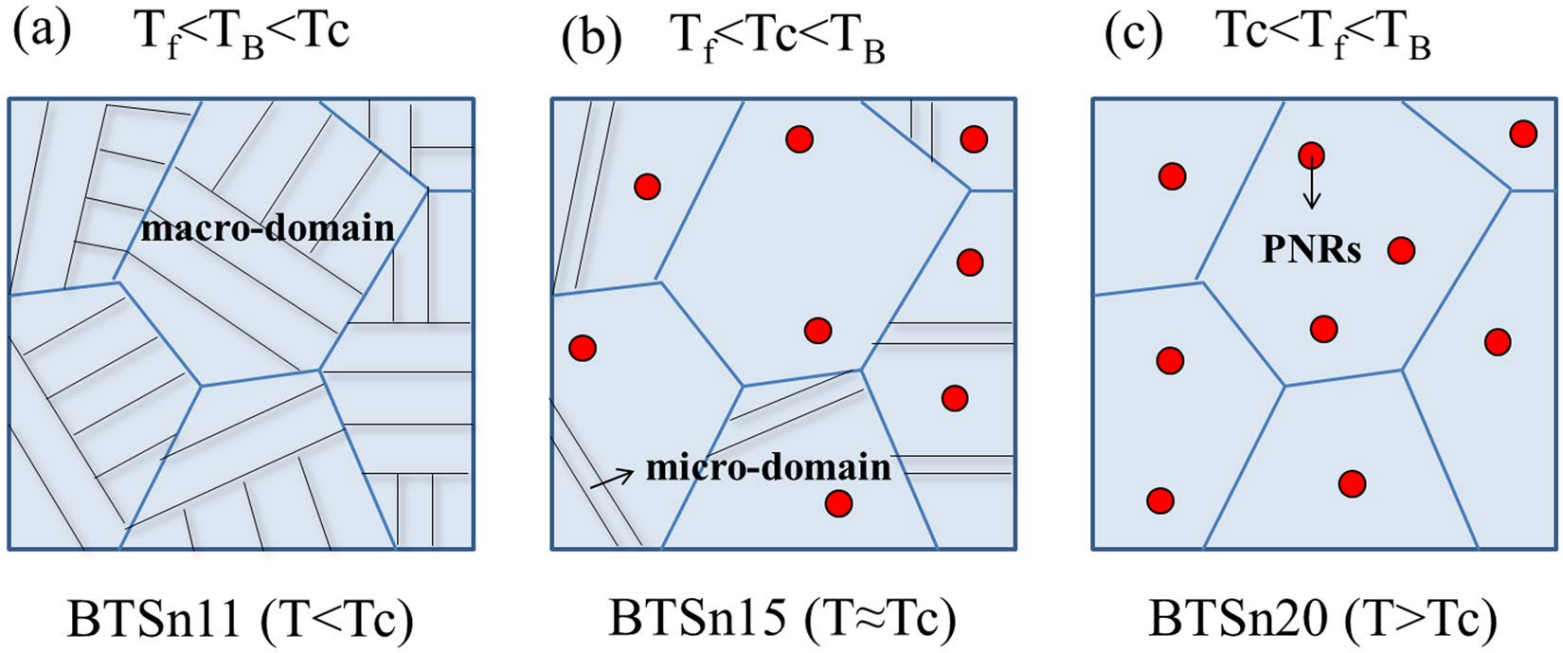
Schematic evolution of domain structures with the increasing of tin concentrations at the measured temperature *T*: (**a**) BTSn11, (**b**) BTSn15 and (**c**) BTSn20.

From the above results, it can be found that the nonlinear dielectric behavior of BTSn is highly related to its domain structures. Figure 5 gives a schematic evolution of domain structures with increasing Sn^4+^ concentrations in BTSn at room temperature. The formation of PNRs is associated with the concentration of tin ions. The PNRs appear within a temperature range, where the upper limit temperature is usually noted as the Burns temperature (*T*_B_) and the lower limit temperature is called the freezing temperature (*T*_f_). If *T*_B_ lies below *T*c, PNRs are not present and the sample is dominated by macro-domains in a ferroelectric state. This is observed for BTSn11 at room temperature and is shown in Fig. 5(a). Dynamic PNRs can only be observed at sufficient tin concentrations. However, if *T*_f_ is below *T*c, the sample resides in ferroelectric long-range ordered state below *T*c, while nanodomains accompanied by PNRs are present above *T*c. This mechanism is likely realized in BTSn15 and is shown in Fig. 5(b). If *T*c lies below *T*_f_, which is probably the case for BTSn20, there will be a transition into a short-range ordered cluster glass state, as shown in Fig. 5(c) ^10^.

**Figure 6:**
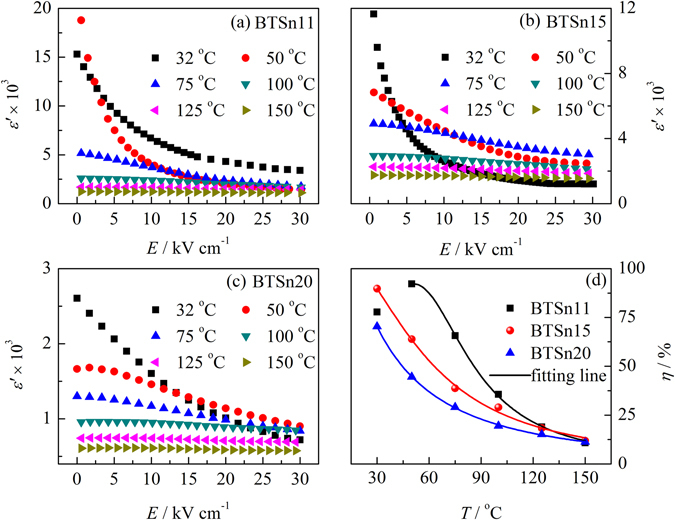
(**a–c**) Dielectric permittivity as a function of *dc* electric field of BTSn11, BTSn15 and BTSn20 from bias electric field measurement taken at 10 kHz and at different temperatures; (**d**) dielectric tunability of BTSn11, BTSn15 and BTSn20 at different temperatures.

Figure 6 (a–c) shows dielectric permittivity as a function of dc electric field of BTSn11, BTSn15 and BTSn20 at 10 kHz and at different temperatures. And the tunability of BTSn11, BTSn15 and BTSn20 at different temperatures is summarized in Fig. 6(d). Tunability of BTSn11 first increases and reaches a maximum value around *T*c, and then gradually decreases. Tunability of BTSn15 and BTSn20 gradually decreases with temperature increasing. Temperature dependent tunability of BTSn also presents a dispersed behavior. In order to evaluate the dispersion of tunability, temperature dependent tunability was also fitted by a Lorenz-type formula:

2 $${\eta }_{A}/\eta =1+{(T-{T}_{A})}^{2}/2{\delta }_{\eta }^{2}$$

where *η*_A_ is a constant and *δ*_*η*_ is the diffuseness of the tunability as a function of the temperature. The fitted *δ*_*η*_ for BTSn11, BTSn15 and BTSn20 are 25.7(1), 40.7(1) and 49.2(1), respectively. The variance of *δ*_*η*_ with the temperature has a similar trend with *δ*, indicating that tunability is also affected by the concentration of Sn ions. Large concentration of Sn ions not only leads to the depression of the dielectric peak at *T*c, tunability is also dispersed due to compositional fluctuation at a local region.

**Figure 7:**
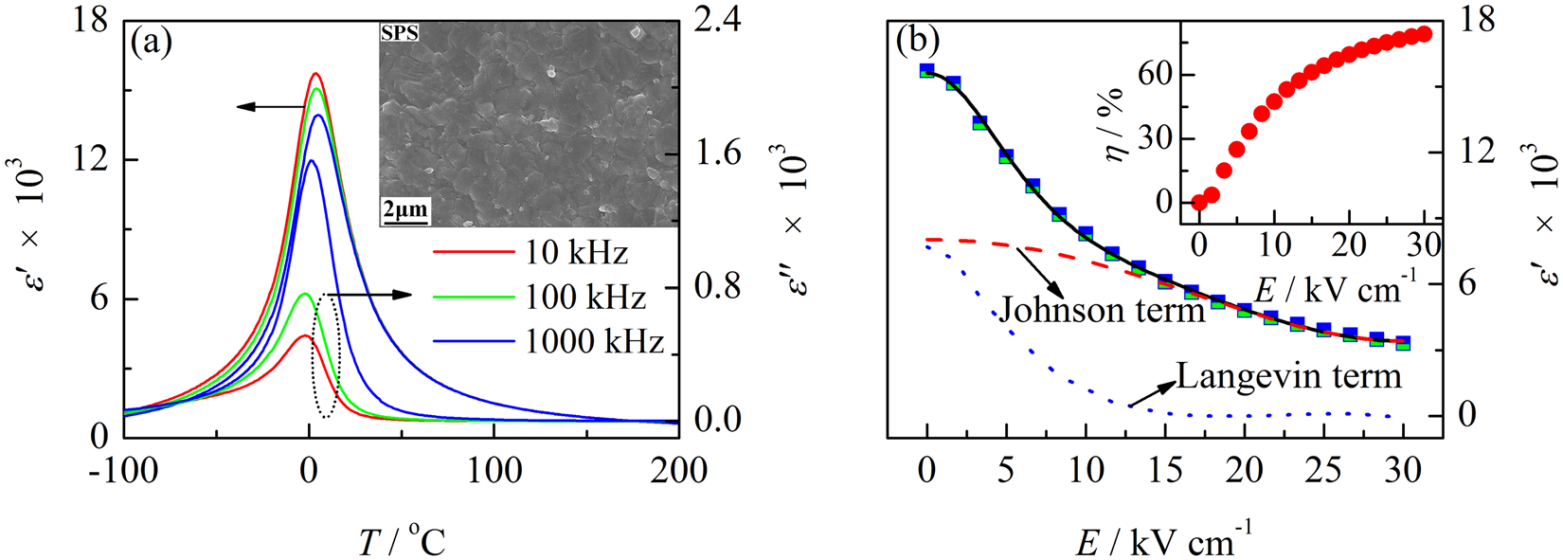
The temperature-dependent real and imaginary parts of dielectric permittivity of BTSn15 prepared by SPS. The inset in Fig. 7(a) shows the SEM image of BTSn15 prepared by SPS; (**b**) the dielectric permittivity as a function of the electric field of BTSn15 prepared by SPS. The inset in Fig. 7(b) shows the tunability of BTSn15 prepared by SPS as a function of electric field.

Besides temperature, the grain size also has some effects on the nonlinear dielectric behavior of BTSn ceramics. BTSn15 ceramics were prepared by an SPS method (labeled as BTSn15-SPS) with a relative density of 98%. Figure 7(a) shows the temperature-dependent real and imaginary dielectric permittivity of BTSn15-SPS. The inset in Fig. 7(a) shows the SEM image of BTSn15-SPS with a grain size less than 1 μm. Dielectric permittivity of BTSn15-SPS is slightly lower than BTSn15 prepared by CS. *dc* bias dependent dielectric permittivity of BTSn15-SPS is also investigated and is shown in Fig. 7(b). The data were also fitted by Eq. (4). The results suggest that the contribution of the Langevin term of BTSn15-SPS is slightly lower than that of BTSn15 prepared by CS. And *L* and *P*_0_ are 5.5(1) nm and 4.1(1) μC/cm^2^. The increase of the polarization might be due to the enhancement of the density of sample. The inset of Fig. 7(b) illustrates the tunability of BTSn15-SPS as a function of the electric field. The maximum tunability is 78.1% at the electric field of 30 kV/cm, which is smaller than that of conventional-sintered BTSn15. The reason is speculated that because BTSn15-SPS has smaller grain size, the rotation of the micro-domain might be hindered by the grain boundary, and ultimately leading to the decrease in tunability and the contribution of Langevin term. This will be studied in detail and the corresponding evidence will be provided in our follow-up work.

## Conclusions

BaTi_1−x_Sn_x_O_3_ (BTSn, 0 ≤ x ≤ 0.30) ceramics that have a wide compositional range, from normal ferroelectricity to the DPT state, were prepared by both the conventional sintering and sparking plasma sintering. BTSn15 was found to have the maximum tunability at the room temperature, which is up to 90.5%. The nonlinear dielectric behavior of BTSn ceramics was analyzed by the multipolarization-mechanism model. The enhanced dielectric tunability is attributed to the presence of mico-domains that coexist with polar nanoregions. This work could provide new insights into the nonlinear dielectric behavior of Pb-free dielectrics.
